# Baihu renshen decoction ameliorates type 2 diabetes mellitus in rats through affecting gut microbiota enhancing gut permeability and inhibiting TLR4/NF-κB-mediated inflammatory response

**DOI:** 10.3389/fcimb.2022.1051962

**Published:** 2022-11-11

**Authors:** Bin Yao, Baochao Pan, Tian Tian, Xiuhai Su, Shufang Zhang, Hanzhou Li, Wendong Li, Yuansong Wang, Shuquan Lv, Zhaiyi Zhang

**Affiliations:** ^1^ Department of Endocrinology, Cangzhou Hospital of Integrated Traditional Chinese Medicine and Western Medicine of Hebei Province Affiliated to Hebei University of Chinese Medicine, Cangzhou, China; ^2^ Graduate School, Chengde Medical University, Chengde, China; ^3^ College of Integrated Traditional Chinese and Western Medicine, Tianjin University of Traditional Chinese Medicine, Tianjin, China

**Keywords:** baihu rensheng decoction, type 2 diabetes mellitus, gut permeability, TLR4/NF-κBmediated inflammatory response, gut microbiota

## Abstract

Baihu Rensheng decoction (BHRS) can effectively improve insulin resistance (IR) and decrease blood glucose in diabetic patients. However, its specific mechanism of action remains unclear. In this study, a type 2 diabetes mellitus (T2DM) rat model was established using a high-fat diet combined with streptozotocin (STZ) injection and treated with BHRS. Firstly, the therapeutic and anti-inflammatory effects of BHRS on T2DM were evaluated. Secondly, the effects of BHRS on gut permeability were evaluated and western blot was used to detect the changes of TLR4/NF-κB pathway-related protein expressions in liver. Finally, 16S rRNA sequencing was used to detect alteration of gut microbiota diversity and abundance in rats after BHRS treatment. Our results showed that BHRS could alleviate the hyperglycemia, hyperlipidemia, IR, and pathological changes of liver, pancreas, and kidney in T2DM rats. BHRS could also decrease the levels of pro-inflammatory cytokines and inhibit the oxidative stress. Immunohistochemistry showed BHRS could increase the expression tight junction-related proteins (ZO-1 and occludin) in colon. Besides, the level of LPS in serum was decreased after BHRS treatment. Western blot results showed that the protein expression of TLR4, MyD88 and the phosphorylation IκB, and NF-κBp65 were lowered after BHRS treatment. 16S rRNA sequencing showed that BHRS treatment altered the diversity of gut microbiotra and decreases the *Firmicutes*/*Bacteroidetes* (*F* to *B*) ratio at the phylum level. At the genus level, BHRS could increase the relative abundances of *Lactobacillus, Blautia*, and *Anaerostipes* and decrease the relative abundances of *Allobaculum, Candidatus Saccharimonas*, and *Ruminococcus*. In conclusion, our study revealed the various ameliorative effects of BHRS on T2DM, including improving the liver and kidney functions and alleviating the hyperglycemia, hyperlipidemia, pathological changes, oxidative stress and inflammatory response. The mechanisms of BHRS on T2DM are likely linked to the repair of gut barrier and the inhibition of TLR4/NF-κB-mediated inflammatory response and the improvement in the dysbiosis of gut microbiota.

## Introduction

Diabetes mellitus (DM) is a chronic systemic metabolic disease characterized by hyperglycemia and is caused by defective insulin secretion and/or insulin resistance (IR). According to the International Diabetes Federation, approximately 463 million people worldwide were diagnosed with diabetes in 2019 and this number is expected to reach 700 million by 2045 (Saeedi et al., 2019). Among these patients, type 2 diabetes mellitus (T2DM) accounts for >90% cases of DM; the main clinical symptoms of which are polydipsia and polyuria. Type 2 diabetes is often complicated by blindness and paralysis, which seriously affect the patients’ quality of life (Chatterjee et al., 2017).

Gut microbiota are important for the health of the host and have functions including defense against pathogen invasion, promotion of substance metabolism, vitamin synthesis and antitumor effects (Schoeler and Caesar, 2019). In-depth studies on T2DM have revealed that gut microbiota dysbiosis is closely related to the development of T2DM (Karlsson et al., 2013). Clinical studies in T2DM have reported that patients have high levels of *Bifidobacteria* sp. in their gut microecology (Sedighi et al., 2017). Another study found that patients with T2DM show a considerable difference in the composition of butyric acid producing bacteria, such as *Lactobacillus* sp., and *Akkermansia muciniphila* in their gut microecology (Aw and Fukuda, 2017). A study also reported low abundance of *Clostridium* sp. in European women with T2DM, where *Clostridium* sp. is negatively associated with fasting blood glucose, glycated hemoglobin, and insulin (Karlsson et al., 2013). These studies suggested that the composition of the gut microbiota is altered in patients with T2DM. Therefore, gut microbiota can be an important target for the prevention and treatment of T2DM, and this fact has been reported in related studies (Brunkwall and Orho-Melander, 2017). Metformin, an oral drug commonly used to treat T2DM, reportedly improved blood glucose in patients with T2DM by regulating gut microbiota (Wu et al., 2017). In patients treated with liraglutide, gut microbial α-diversity was reduced, and KEGG prediction analysis revealed that liraglutide may act through phenylalanine, porphyrin and chlorophyll, and selenocompound metabolic pathways (Shang et al., 2021).

Traditional Chinese medicine (TCM) is widely used in various metabolic diseases and has a positive effect on the regulation of gut microbiota (Cao et al., 2022). Berberine is an alkaloid isolated from *Coptis chinensis* Franch., which can effectively increase the variety of probiotics in the intestinal tract, reduce the distribution of harmful bacteria, and reduce the inflammatory response and endotoxemia. Furthermore, it is known to improve obesity and IR and has good efficacy in T2DM (Zhang et al., 2012). *Polygonatum sibiricum* Redouté polysaccharide significantly reduced the count of *Ruminococcus* sp. in the intestine of rats, while increasing the distribution and count of the *Bacillus* sp., which has a bidirectional regulatory effect on blood glucose and gut microbiota in T2DM rats and positively improved abnormal glucose metabolism (Yan et al., 2017). Huanglianjiedu Decoction significantly improved hyperglycemia and inflammatory symptoms in T2DM rats by increasing the number of short-chain fatty acids (SCFAs) and anti-inflammatory bacteria (Chen et al., 2018).

Baihu Rensheng decoction (BHRS) consists of *Anemarrhena asphodeloides* Bunge, gypsum, *Glycyrrhiza glabra* L., Japonica rice, and *Panax ginseng* C.A.Mey. The role of BHRS in the treatment of diabetes first appeared in Zhang Zhongjing’s Treatise on *Shanghan Lun*, with the effects of dispelling, invigorating qi and promoting the secretion of body fluids (Tian et al., 2020). Clinically, BHRS can effectively improve IR in diabetic patients, thereby improving insulin sensitivity and lowering blood lipids and blood glucose (Zhao, 2018). However, its specific mechanism of action remains unclear.

In this study, a type 2 diabetes mellitus (T2DM) rat model was established using a high-fat diet combined with streptozotocin (STZ) injection and treated with BHRS. Firstly, the therapeutic and anti-inflammatory effects of BHRS on T2DM were evaluated. Secondly, the effects of BHRS on gut permeability were evaluated and western blot was used to detect the changes of TLR4/NF-κB pathway-related protein expressions in liver. Finally, 16S rRNA sequencing was used to detect alteration of gut microbiota diversity and abundance in rats after BHRS treatment. This can help to clarify the mechanism of action of BHRS in alleviating IR in T2DM by regulating gut microbiota, improving intestinal mucosal permeability, and inhibiting the activation of TLR4/NF-κB signaling pathway.

## Materials and methods

### Reagents

The reagents used in this study were shown in **Supplementary Materials**.

### Animals

Fifty male Sprague Dawley (SD) rats, weighing 200 ± 20 g, were purchased from Beijing HFK Bioscience Co., Ltd. (SCXK 2021-0007). The housing environment was maintained at 25°C ± 2°C, relative humidity was 50% ± 15%, light-dark cycle was 12h/12h, and animals were given ad libitum access to food and water. The animal study was approved by Ethics Committee of Hebei University of Chinese Medicine (Approval no. CZX2021-KY-026).

### Generation of the T2DM rat model

T2DM was induced in rats as described previously (Xie et al., 2021). Briefly, rats were fed with high-fat diet (maintenance feed 65% + lard 10% + sucrose 20% + cholesterol 2.5% + sodium cholate 1% + mixer 1.5%) for 4 weeks after 7 d of acclimatization, and 35 mg/kg 1% STZ sodium citrate buffer was injected intraperitoneally. After 3 d of overnight fasting, fasting blood was collected from the tail vein every 24 h to measure fasting blood glucose, and generation of the diabetic rat model was considered successful when blood glucose ≥16.7 mmol/L for 3 consecutive days.

### Preparation of BHRS extractions

An 18g of *Anemarrhena asphodeloides* Bunge, 40g of gypsum (crushed, wrapped in cotton), 6g of *Glycyrrhiza glabra* L., 15g of japonica rice, and 9g of *Panax ginseng* C.A.Mey. were weighed, 8 volumes of water were added and decocted for 30 min twice. The BHRS extractions obtained from two-time of decoction were mixed and filtered. Then, the BHRS extraction was concentrated to 0.88g of crude drugs/and 1.76 g of crude drugs/mL respectively for subsequent experiments. For the quality control of BHRS, ultra performance liquid chromatography (UPLC) coupled with quadrupole-time of-flight (Q-TOF) mass spectrometer (MS) systems were used to identify the main components in BHRS, as shown in the **Supplementary Materials**.

### Grouping and dosing

After 1 week of acclimatization, 10 rats were randomly selected as the control group and fed with normal chow, while the remaining 40 rats were selected to induce the T2DM model. At the end of the modeling period, we divided T2DM rats into T2DM group, metformin group, BHRS low-dose group, and BHRS high-dose group randomly, with 10 rats in each group. Then, the control and T2DM groups were intragastrically administered 1 mL/kg of saline, the rats in metformin group was intragastrically administered with metformin (0.2 g/kg/d) (Radbakhsh et al., 2021), the rats in BHRS low-dose and BHRS high-dose groups was intragastrically administered BHRS extractions 0.88g of crude drugs/mL and 1.76 g of crude drugs/mL respectively, once/d, all for 4 weeks. The dosing volunm of BHRS extraction was 1mL/kg. Fasting blood glucose (FBG) of rats in each group was measured weekly. The dose setting of BHRS was based on the equivalent dose conversion formula. BHRS low-dose group was the human equivalent dose. BHRS high dose group was 2× of low-dose respectively.

### Efficacy observation

#### Oral glucose tolerance test

After 4 weeks of BHRS intervention, all rats were fasted food without fasting water for 8 h. Blood glucose level was detected using blood glucose testing strip as the blood glucose value at 0 min, and then each rat was immediately given 50% glucose solution (2 g/kg) *via* intragastric administration, and the blood glucose value was measured after giving glucose solution for 15, 30, 60,and 120 min, respectively, where the blood glucose-time curve was plotted to calculate the area under the curve (AUC) of OGTT.

### Serum biochemical marker test

After 4 weeks of intervention with BHRS, all rats were fasted without water restriction for 8 h. Rats were anesthetized by intraperitoneal injection of sodium pentobarbital (50 mg/kg), blood was collected from the abdominal aorta. The collected blood was centrifuged at 3000 r-min^−1^ for 15 min to collect the serum. Assay kits were used to detect the levels of lipid-related markers (triglyceride (TG), total cholesterol (TC), high-density lipoprotein (HDL), low-density lipoprotein (LDL)), liver function-related markers (alanine aminotransferase (ALT) and aspartate aminotransferase (AST)), kidney function-related markers (creatinine (Cr) and blood urea nitrogen (BUN)), oxidative stress-related markers (superoxide dismutase (SOD) and plasma glutathione peroxidase (GSH-Px) activities and malondialdehyde (MDA) and levels in the serum of rats in each group, and the procedure was performed according to the description of the kit.

### Enzyme linked immunosorbent assay

After 4 weeks of BHRS intervention, serum levels of insulin, interleukin (IL)-6, IL-1β, tumor necrosis factor (TNF)-α and lipopolysaccharide (LPS) were measured in each group using ELISA, which was performed according to the steps described in the kit. The homeostatic model assessment for IR (HOMA-IR) was calculated with the following formula: HOMA-IR = (fasting glucose x fasting insulin)/22.5.

### Pathology staining

After collecting the serum, rat liver, pancreas and kidney tissues were fixed with formalin solution, paraffin embedded, made into 3 μm sections, routinely stained with hematoxylin and eosin (HE), and each histopathological change was observed under a light microscope.

### Immunohistochemistry

The colon tissues were fixed with formalin solution, paraffin embedded, made into 3 μm sections and the expression of ZO-1 (1:200) and occludin (1:300) protein in colon was detected by immunohistochemistry as described previously (Liao et al., 2021). The positive area for ZO-1 and occludin staining was quantified using Image Pro Plus 6.0 software based on the average optical density (AOD).

### Western blot

20 mg of liver tissues were weighed and added to 150 μL of RIPA protein lysis buffer to isolate the protein. Total protein concentration was determined using the BCA protein assay kit. Equal amounts of 10 μg of protein were taken from each sample, and the proteins were resolved using SDS-PAGE electrophoresis under the following conditions: 90 V, 20 min; 130 V, 1 h. The separated proteins were then transferred to PVDF membrane at 130V, 300 mA, 2h, blocked with 5% skim milk powder at room temperature for 2 h. Different rabbit anti-rat primary antibodies TLR4, MyD88, IκB, p-IκB, NF-κBp65, p-NF-κBp65, and β-action at dilution ratios of 1:1000, 1: 1000, 1:1000, 1:1000, 1:1000, 1:1000, 1:1000, and 1:2000, respectively were added and the membranes were incubated overnight at 4°C. After washing the membrane, secondary antibody (goat anti-rabbit IgG diluted at 1:8,000) was added and incubated at room temperature for 2 h. After washing the membrane with tris-buffered saline (TBST), enhanced chemiluminescence (ECL) was added to develop and detect the bands with image pro plus 6.0 software for quantitative analysis of the grayscale values.

### Quantitative polymerase chain reaction

Briefly, the total RNA in liver tissue was isolated using the RNA isolation kits. Then, the quality and concentration of RNA sample was detected using nanodrop. RNA samples were diluted into same concentration and reversely transcribed into cDNA based on the steps described in the kit. PCR amplification was performed using cDNA as a template. The primers and SuperReal PreMix Plus were added according to the kit instructions. The primer sequences were shown in **Table S2**. *β-actin* was used as a loading control. The relative expression of *IL-1β, IL-6* and *TNF-α* was calculated based on 2^-△△CT^ protocol.

### 16S rRNA sequencing

#### Fecal genomic DNA extraction

The total DNA of rat cecal contents was extracted by using CTAB/SDS method, and the DNA concentration and purity were measured using 1% agarose gel. DNA was diluted to 1 ng/µL with sterile water, depending on the concentration.

#### PCR amplification and sequencing of 16S rRNA gene

The primers 338F (5′-ACTCCTACGGGAGGCAGCAG-3′) and 806R (5′-GGACTACHVGGGTWTCTAAT-3′) were used to amplify the V3 to V4 regions of the 16S rRNA gene. The PCR amplification system includes the following: 10 ng of template DNA, 0.2 µM of forward and reverse primers, 15 µL Phusion^®^ High-Fidelity PCR Master Mix (New England Biolabs). The reaction conditions were as follows: pre-denaturation at 98°C for 1 min; denaturation at 95°C for 10 s, annealing at 50°C for 30 s, and extension at 72°C for 30 s, for a total of 15 cycles; and finally holding at 72°C for 5 min and storage at 4°C. The mixed PCR products were purified using Qiagen Gel Extraction Kit (Qiagen, Germany). The 2% agarose gel electrophoresis was used for testing. Sequencing libraries were generated using TruSeq^®^ DNA PCR-Free Sample Preparation Kit (Illumina, USA) and library quality was assessed by Qubit@ 2.0 Fluorometer (Thermo Scientific) and Agilent Bioanalyzer 2100 system. Finally, the library was sequenced on the Illumina NovaSeq platform to obtain 250 bp of paired-end sequences.

#### Sequencing data analysis

The raw sequencing data were assembled using FLASH (V1.2.7, http://ccb.jhu.edu/software/FLASH/) and final effective tags were obtained after quality control. The Tags were clustered using Uparse software (Uparse v7.0.1001, http://drive5.com/uparse/) at 97% similarity level to obtain the operational taxonomic units (OTUs). Mothur algorithm-based Silva database (http://www.arb-silva.de/) was used for taxonomic information annotation of OTUs. MUSCLE software (Version 3.8.31, http://www.drive5.com/muscle/) was used to perform multiple sequence comparison. OTUs abundance information was normalized by the sequence number corresponding to the sample with the smallest sequence. Alpha diversity index and beta diversity analysis were performed subsequently. Alpha diversity refers to the average species diversity in an area of the environment. Shannon and Simpon indices were used to assess the alpha diversity of gut microbiota. Besides, the beta diversity was presented by principal coordinate analysis plot (PCoA) analysis and clustering analysis. The Wilcoxon rank-sum test was used to test for differences between groups in diversity indices. The Kruskal–Wallis rank-sum test (Games-Howell was chosen as the *post-hoc* test combined with the multiple testing method FDR was used to screen for differential bacteria, and a difference with *P* < 0.05 was considered to be a significant.

### Statistical analysis

Statistical analysis was performed using SPSS 20.0 statistical software, and data was expressed as mean ± standard deviation. One-way analysis of variance (ANOVA) followed by Tukey’s *post-hoc* analysis was used for comparison among groups, A difference with P < 0.05 was considered to be statistically significant.

## Results

### BHRS treatment improved T2DM in rats

**Figure 1:**
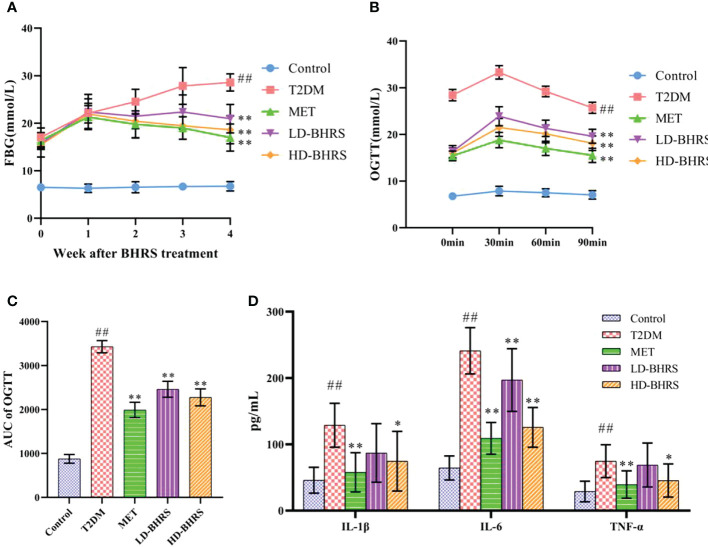
BHRS treatment decreased the levels of fasting blood glucose (FBG), lowered the area under the curve (AUC) of oral glucose tolerance test (OGTT) and reduced the serum levels of pro-inflammatory cytokines (IL-1β, IL-6 and TNF-α). **(A)** FBG of each group. **(B, C)** OGTT and OGTT-AUC of each group. **(D)** Serum pro-inflammatory cytokines of each group. ^##^: *p* < 0.01 as compared to the control group; *: *p* < 0.05 as compared to the T2DM group; **: *p* < 0.01 as compared to the T2DM group.

The blood glucose of the rats was higher than 16.7 mmol/L 72 hours after STZ injection. Then, after BHRS treatment, the FBG levels in the MET, LD-BHRS, and HD-BHRS groups were significantly decreased compared to those in the T2DM group (**Figure 1A**). The OGTT results showed that the OGTT-AUC was significantly higher in the T2DM group than in the Control group, whereas the OGTT-AUC was lower in the MET, LD-BHRS and HD-BHRS groups than in the T2DM group (**Figures 1B, C**). The changes in blood lipid levels were detected. Compared to the Control group, serum levels of TG, TC, and LDL were significantly higher in the T2DM group, whereas HDL levels were relatively lower. Compared to the T2DM group, the MET and BHRS treatment groups had decreased TC, TG, and LDL levels and increased HDL levels. In terms of hepatic and renal function, the activities of serum ALT, AST, Cr, and BUN were significantly increased in the T2DM model rats compared to the Control group, and these parameters were significantly decreased in the MET and BHRS treatment groups compared to those in the T2DM group. In addition, FINS level and HOMA-IR value were significantly increased in the T2DM group compared with the control group. However, these levels were lower in the MET, LD-BHRS, and HD-BHRS groups those in the T2DM group (**Table 1**).

**Table 1:** Changes in physiological indices, oxidative stress factors, and insulin resistance after BHRS treatment.

	Biochemical Parameters	Control	T2DM	MET	LD-BHRS	HD-BHRS
**Blood lipid profile**	TC (mmol/L)	12±4.2	22.2±7.4^##^	13.5±5.7^*^	16.3±5.4	15.1±5.2^*^
	TG (mmol/L)	2.3±0.5	5.9±0.6^##^	2.6±0.9^**^	3.7±1.0^**^	2.8±0.8^**^
	HDL (mmol/L)	6.9±1.6	3.7±1.2^##^	6±1.7^**^	4.8±1.3	5.4±1.2^**^
	LDL (mmol/L)	1.8±0.4	4.9±0.8^##^	2.5±0.8^**^	3.2±1.5^**^	2.7±0.6^**^
**Liver and kidney function**	ALT (U/L)	29.2±13.1	141.7±25.4^##^	59.9±22.3^**^	88.2±48.0^**^	71.3±30.2^**^
	AST (U/L)	62.5±26.2	127.8±45.5^##^	71.0±39.6^**^	106.6±47.7	81.0±28.8^*^
	Cr (μmol/L)	39.7±15.1	125.6±26.2^##^	47.4±19.2^**^	60.4±26.7^**^	56.8±21.8^**^
	BUN (mmol/L)	5.5±2.6	11.8±2.9^##^	6.7±1.6^**^	9.4±3.1	7.1±1.8^**^
**Insulin resistance**	FINS (μIU/mL)	6.4±2.6	15.7±4.5^##^	6.5±2.3^**^	9.9±5.9^*^	7.0±2.8^**^
	HOMA-IR	1.9±0.7	23.1±4.0^##^	4.6±0.9^**^	11.9±4.1^**^	4.9±1.6^**^
**Oxidative stress**	SOD (U/mL)	197.8±17.8	110.2±18.6^##^	170.7±28.7^**^	137.9±24.6^*^	167.2±25.4^**^
	MDA (nmol/mL)	4.7±0.8	19.5±2.1^##^	11.2±2.1^**^	13.7±2.2^**^	13.0±1.7^**^
	GSH-Px(μmol/L)	88.7±9.0	56.7±12.2^##^	80.5±16.3^**^	72.1±17.4^*^	76.9±19.6^*^

Control, T2DM, MET, LD-BHRS and HD-BHRS (n = 10 per group) groups. Data are presented as the mean ± SD. ^##^: p < 0.01 as compared to the control group; ^*^: p < 0.05 as compared to the T2DM group; ^**^: p < 0.01 as compared to the T2DM group.

**Figure 2:**
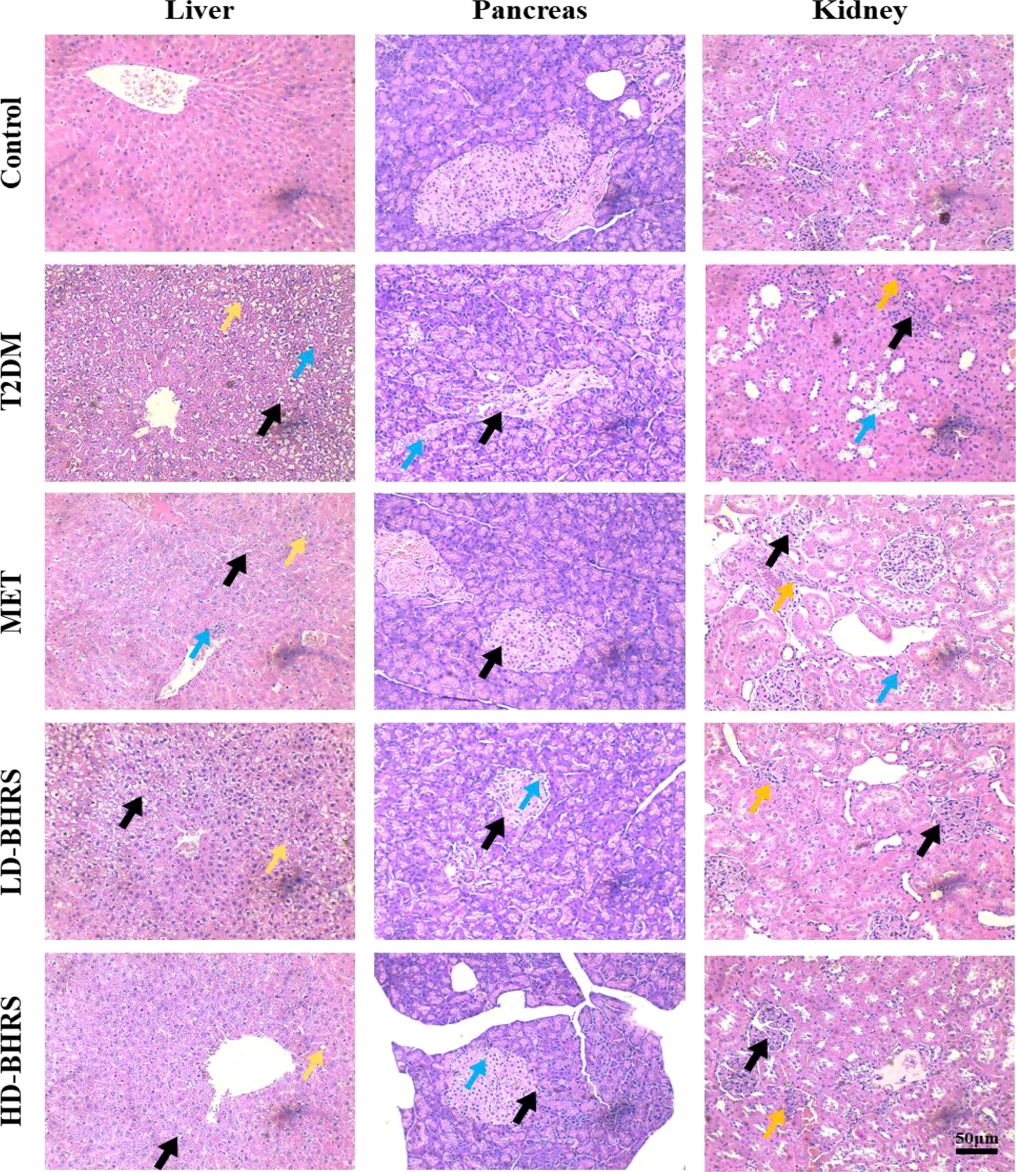
HE staining indicated that the pathological changes of liver, pancreas, and kidney were reduced in T2DM model rats after BHRS treatment (Liver: black arrows indicated the cellular swelling of hepatocytes, yellow arrows indicated the steatosis of hepatocytes, and blue arrows indicated the infiltration of inflammatory cells; Pancreas: black arrows indicated the impaired structure of pancreatic islets, and blue arrows indicated the vacuole-like deformation in endocrine cells; Kidney: black arrows indicated the glomerular hyperplasia, blue arrows indicated the atrophy of the renal tubules, and yellow arrows indicated the inflammatory cell infiltration) (200×).

The pathological changes in liver, pancreas, kidney and colon in T2DM rats after BHRS treatment were also observed by HE staining. The liver pathological results showed that in the T2DM group hepatic lobules were poorly defined, with swollen hepatocytes, loose and lightly-stained cytoplasm, reduced density, and irregular morphology, as well as inflammatory cells were locally infiltrated. The pancreas pathological results showed that in the T2DM group the pancreatic islets of T2DM rats were significantly smaller in size, sparsely distributed, with blurred boundaries with the surrounding area; the endocrine cells showed vacuole-like deformation. The kidney pathological results showed that in the T2DM group apparent atrophy of the renal tubules, and glomerular hyperplasia with a certain degree of inflammatory cell infiltration. Intervention with metformin and BHRS significantly improved the histopathological changes in the liver, pancreas, and kidney of rats in the T2DM model (**Figure 2**).

### BHRS treatment improved oxidative stress and inflammatory response in T2DM rats

We also evaluated the effect of BHRS on oxidative stress in T2DM rats. Compared to the Control group, the activities of SOD and GSH-Px in the serum of rats in the T2DM group were significantly decreased and the levels of MDA were significantly increased. SOD and GSH-Px activities were elevated in the MET and BHRS treatment groups compared to those in the T2DM group, whereas MDA levels were decreased in the MET and BHRS treatment groups compared to those in the T2DM group (**Table 1**).

In addition, to investigate the effect of BHRS on the inflammatory response in T2DM rats, we measured the levels of pro-inflammatory cytokines (IL-1β, IL-6, and TNF-α) in the serum. The serum levels of IL-1β, IL-6, and TNF-α were significantly increased in T2DM rats compared to those in the Control group. Metformin and high-dose of BHRS treatment decreased the levels of IL-1β, IL-6, and TNF-α in T2DM model rats. IL-6 levels also appeared to decrease in the LD-BHRS group compared with that in the T2DM group (**Figure 1D**).

The above results support the therapeutic effect of BHRS in T2DM rats, which was most evident at high doses. Therefore, the HD-BHRS group was selected for the follow-up mechanic studies.

### BHRS treatment increased the gut permeability and reduced endotoxemia in T2DM model rats

**Figure 3:**
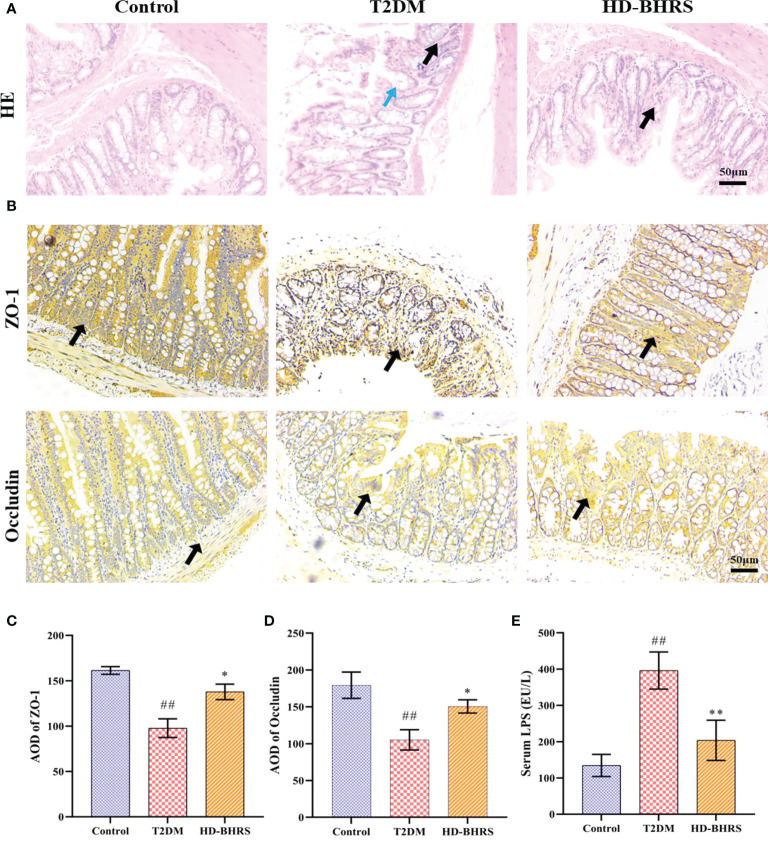
BHRS treatment reduced the pathological changes of colon and increased the expression tight junction proteins (ZO-1 and Occludin) in colon and decreased the level of lipopolysaccharide (LPS) in serum. **(A)** HE staining indicated that the pathological changes of colon (Black arrows indicated the infiltration of inflammatory cells, blue arrows indicated the impaired structure of colonic epithelial cell) (200×). **(B–D)** Immunohistochemistry was used to analysis the expression of ZO-1 and Occludin in colon (200×) and the average optical density (AOD) of positive area was calculated using Image Pro Plus 6.0. **(E)** The LPS level in serum was tested using enzyme linked immunosorbent assay (ELISA). ^##^: *p* < 0.01 as compared to the control group; *: *p* < 0.05 as compared to the T2DM group; **: *p* < 0.01 as compared to the T2DM group.

T2DM is accompanied by the impair of gut barrier. The impaired gut barrier could cause the decrease of gut permeability and trigger endotoxemia (the LPS of gut microbiota could enter periphery through lack gut barrier) (Nascimento et al., 2020). We further tested the effects of BHRS on gut permeability by measuring the expression of tight junction protein ZO-1 and occludin in colon. The colon pathological results showed that the morphology of epithelial cells was disrupted and inflammatory cell infiltration can be observed in colon in the T2DM group whereas high-dose of BHRS treatment improved the pathological changes in colon in T2DM model rats (**Figure 3A**). Immunohistochemical results showed that the area of positive expression of ZO-1 and occludin was significantly reduced in the colonic tissue of rats in the T2DM group compared to those in the Control group. After receiving HD-BHRS treatment, the area of ZO-1 and occludin positive expression in the colonic tissue of T2DM rats showed varying degrees of elevation (**Figures 3B–D**)

We also tested the effects of BHRS on endotoxemia by measuring the levels of LPS in serum using ELISA. Results showed that serum LPS levels were significantly increased in T2DM rats compared to those in the Control group, while there was some decrease after HD-BHRS intervention (**Figure 3E**).

### BHRS treatment inhibited the activation of TLR4/NF-κB signaling pathway

**Figure 4:**
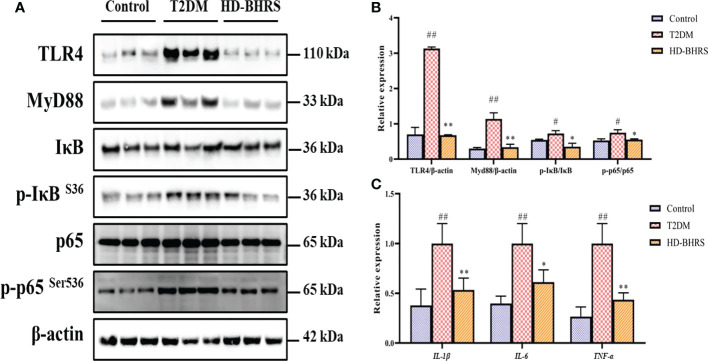
BHRS treatment inhibited the activation of TLR4/NF-κB signaling pathway. **(A, B)** The protein levels of TLR4 and MyD88 and the phosphorylation of IκB, and NF-κBp65 in liver were detected using western blot and the relative expressions of proteins were quantified based on grayscale values using Image J analysis software. β-actin was used as the loading control. For detecting the phosphorylation of proteins, the relative expression was calculated by the specific value of phosphorylated protein to total protein. **(C)** The gene expression of pro-inflammatory cytokines (*IL-1β, IL-6* and *TNF-α*) in liver were detected using qPCR. ^#^: *p* < 0.05 as compared to the control group; ^##^: *p* < 0.01 as compared to the control group; *: *p* < 0.05 as compared to the T2DM group; **: *p* < 0.01 as compared to the T2DM group.

After entering the periphery, the LPS could cause inflammatory response through activating TLR4/NF-κB signaling pathway. Liver tissues were collected from each group of rats, and the levels of proteins related to TLR4 signaling pathway (TLR4, MyD88, IκB, p-IκB, NF-κBp65, and p-NF-κBp65) were detected by Western blot. Results showed that the expression of TLR4 and MyD88 and the phosphorylation of IκB, and NF-κBp65 were significantly higher in the liver of rats in the T2DM group compared to those in the Control group, while the expression of all the above proteins was decreased after HD-BHRS treatment (**Figures 4A, B**). Moreover, the gene expression of *IL-1β, IL-6* and *TNF-α* was up-regulated in T2DM group compared to those in the Control group, while the expression of all the above genes was down-regulated after HD-BHRS treatment (**Figure 4C**).

### BHRS treatment affected the gut microbiota in T2DM model rats

**Figure 5:**
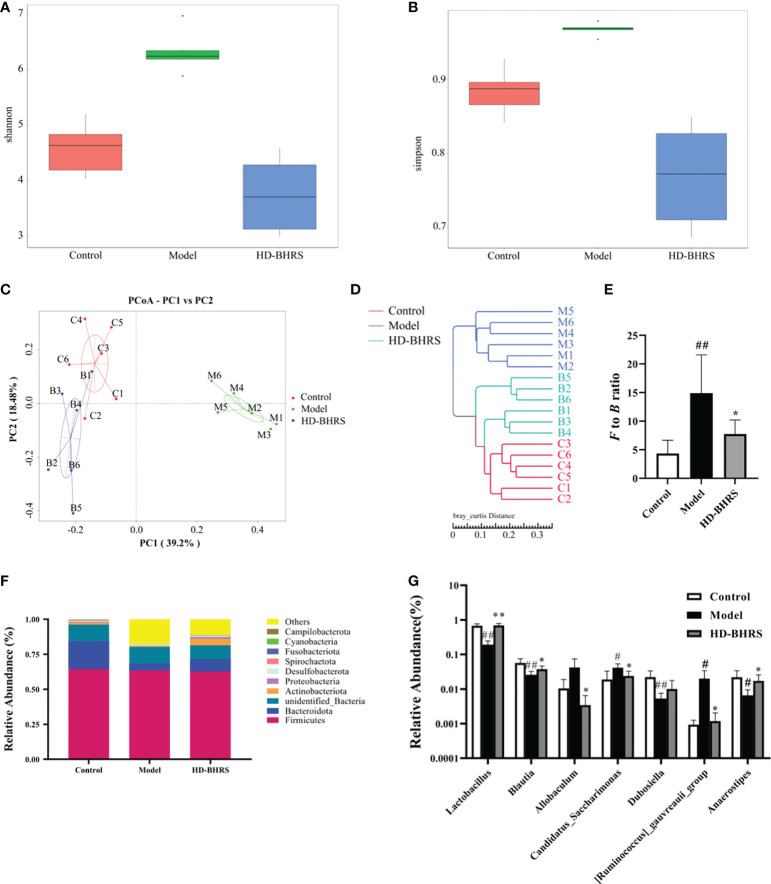
BHRS treatment affected the gut microbiota community in T2DM model rats. **(A, B)** Shannon and Simpson index was lower in HD-BHRS group than that in the T2DM group. **(C, D)** PCoA and system clustering tree indicated more similar beta diversity between HD-BHRS and control groups than that between the T2DM and control groups (C: Control group; T: T2DM group; B: HD-BHRS group). **(E, F)** At the phylum level, BHRS treatment decreased the *F* to *B* ratio in T2DM model rats. **(G)** At the genus level, BHRS treatment affected the relative abundances of *Lactobacillus*, *Blautia*, *Allobaculum, Candidatus_Sacccharimonas, Dubosiella*, *[Ruminococcus]_gauvreauii_group* and *Anaerostipes* in T2DM model rats.

We analyzed the fecal flora of different groups of rats using 16S rRNA high-throughput sequencing. The alpha diversity of the gut microbial community was assessed by calculating the Shannon and Simpson indices. The results showed that the Shannon and Simpson indexes were increased in the T2DM group compared with the control group, and they were decreased in HD-BHRS group compared with the T2DM group (**Figures 5A, B**). Next, we analyzed the differences in beta diversity by principal coordinate analysis (PCoA) and clustering analysis. The PCoA results showed that the samples in the T2DM group were significantly separated from those in the control group, while the samples in the HD-BHRS group were distributed in a similar area to those in the control group (**Figure 5C**). Clustering analysis also showed similar results (**Figure 5D**).

Furthermore, we analyzed the relative abundances of gut microbiota in each group. *Firmicutes* and *Bacteroidetes* were the dominant taxa in gut microbiota at the phylum level for each group. The *Firmicutes/Bacteroidetes* (*F* to *B*) ratio was significantly higher in the T2DM group compared to that in the control group while the *F* to *B* ratio was decreased after high-dose of BHRS intervention (**Figures 5E, F**). At the genus level, the relative abundances of *Candidatus Saccharimonas* and *Ruminococcus* were significantly higher, and the relative abundances of *Lactobacillus, Blautia, Dubosiella*, and *Anaerostipes* were significantly lower in the T2DM model compared to those in the control group; compared to the T2DM group, the relative abundances of *Lactobacillus, Blautia*, and *Anaerostipes* were significantly increased, and the relative abundances of *Allobaculum, Candidatus Saccharimonas*, and *Ruminococcus* significantly decreased in the HD-BHRS group (**Figure 5G**).

## Discussion

In this study, we used high-fat chow combined with STZ injection to establish a T2DM rat model. Many of the hallmark pathophysiological changes such as significant increases in blood glucose and lipid levels, lipoprotein disorders, abnormal biochemical parameters related to liver and kidney function, increased IR were observed as expected. All these manifestations are consistent with previous studies (Hu et al., 2019; Zeng et al., 2019; Elshemy et al., 2021). The pathological results showed that significant hepatocyte steatosis, hepatocyte necrosis and inflammatory cell infiltration in the liver, pancreatic islet atrophy, and islet cell injury in pancreas, glomerular basement membrane hyperplasia, tubular atrophy, and massive inflammatory cell infiltration in the kidney, which were consistent with the pathology of T2DM (Lu et al., 2021; Piccolo et al., 2021; Zhao et al., 2021; Duan et al., 2022).

After BHRS treatment, the blood glucose and lipid levels of T2DM rats decreased, the biochemical markers related to lipoprotein and liver and kidney functions improved, and the histopathological changes of liver, kidney, and pancreas were alleviated. The above results suggest that BHRS has a therapeutic effect on T2DM, and it is most significant in the high-dose group. In addition, we selected metformin as a positive control drug (Rena et al., 2017). The results showed that metformin resulted in significant improvement in blood glucose in T2DM rats and had similar improvements in pathological markers and pathological changes as the high-dose BHRS group. These results suggested that BHRS could be used as an alternative regimen to metformin in the treatment of T2DM.

We also investigated the oxidative stress levels and inflammation-related factors in the serum of rats after BHRS treatment. Our results showed that BHRS significantly reduced the levels of the already elevated pro-inflammatory factors IL-1β, IL-6, and TNF-α in the serum of T2DM rats. Numerous studies have shown that hyperglycemic states can cause chronic inflammation and release large amounts of pro-inflammatory factors such as IL-1β, IL-6, and TNF-α, that chronic inflammation has an important role in the development and progression of vasculopathy and neuropathy in the middle and late stages of diabetes, and that inflammatory factor levels are positively correlated with the severity of T2DM (Hwang et al., 2008; Akash et al., 2013). Besides, BHRS increased the SOD and GSH-Px activities and decreased the MDA level in T2DM model rats, indicating the anti-oxidative potential of BHRS. Oxidative stress is an important pathological manifestation in T2DM. High glucose level in the body could induce the accumulation of reactive oxygen species (ROS) in organs and the ROS could trigger oxidative stress and impair the cell membrane. SOD and GSH-Px are important enzymes participating in eliminating the ROS. MDA is the end-product of lipid peroxidation and the MDA level can reflect the severity of oxidative stress (Xie et al., 2021).

Barrier function is a fundamental role of all epithelial cells and is based on the integrity and contribution of many cytoskeletal and membrane proteins that constitute and regulate the tightly connected complexes that exist between cells (Hull and Staehelin, 1979). Many studies have shown that T2DM, especially advanced T2DM, is strongly associated with loss of colonic barrier integrity (Shan et al., 2013), the loss of colonic barrier integrity could trigger the translocation of gut microbiota and their products such as LPS from gut to periphery and further cause endoxemia (Nascimento et al., 2020). The endoxemia induced by translocation of gut microbiota and their products is an important factor for activating the inflammatory response in T2DM (Macho-González et al., 2021). Therefore, we further tested the effects of BHRS on tight junction in T2DM model rats by measuring the expression of ZO-1 and occludin in colon. Our reults found that BHRS treatment elevated the levels of ZO-1 and occludin in colon. Occludin has been extensively studied and is an integral membrane protein localized in tight junctions, where its localization in the tight junction requires binding to the underlying cytoskeleton *via* ZO-1 (Furuse et al., 1994). In addition to this, ZO-1 has been found to interact extensively with a number of other tight junction-related proteins, including ZO-2, ZO-3, β-catenin, paxillin, talin, and peripheral actin rings (González-Mariscal et al., 2003). Therefore, we chose these two proteins to assess the barrier condition of colonic tissues. As seen from the results, the colonic barrier was severely damaged in T2DM rats, whereas the barrier was somewhat restored after BHRS treatment. Our results also showed that BHRS treatment reduced the LPS level in serum, indicating that BHRS could reduce endoxemia in T2DM rats.

LPS is an important structural component of the outer membrane of Gram-negative bacteria. TLR4 is its important sensor that induces the release of pro-inflammatory cytokines upon stimulation (Poltorak et al., 1998). The TLR4 downstream adaptor MyD88 is a key adaptor in interleukin-1 receptor (IL-1R) signaling, and a study demonstrated that MyD88-deficient mice exhibit resistance to LPS-induced sepsis (Kawai et al., 1999). Further downstream of the MyD88-based signaling pathway, the IKK (IκB kinase) and MAPK pathways are activated (Sato et al., 2005). IKKa, IKKb and IKKc form a complex that phosphorylates IκB. This phosphorylation leads to the degradation of IκB protein and the subsequent translocation of the transcription factor NF-κBp65, which controls the expression of pro-inflammatory cytokines, as well as other immune-related genes (Gasparini and Feldmann, 2012). Based on the above theory, we selected TLR4, MyD88, IκB, p-IκB, and p65 and p-p65, the most common of the NF-κB family, as key markers to explore the inflammation-causing TLR4-NF-κB signaling pathway. The results showed that BHRS treatment decreased the protein levels of TLR4, MyD88 and the phosphoralytions of IκB and p65, suggesting that inhibition of the TLR4/NF-κB signaling pathway is a step in the mechanism of inflammation alleviation by BHRS.

The potential role of gut microbiota in T2DM has been extensively studied and has been shown to be strongly associated with the impair of gut barrier and inflammation during T2DM progression (Cani et al., 2012; Wen and Duffy, 2017; Gurung et al., 2020). We further tested the effects of BHRS on gut microbiota in T2DM model rats. Simpson index and Shannon index are both used to calculate the diversity of microbiota, whereas PCoA and clustering tree reflect the magnitude of the difference of populations between groups. By combining the two results, it can be seen that the gut microbiota of T2DM rats was disrupted, and the diversity of microbiota and the difference between populations were significantly changed, while it was improved after receiving HD-BHRS treatment, and the difference between populations was small and leaning toward the Control group.

At phylum level, the F/B ratio was significantly higher in the T2DM group than in the control group, whereas the HD-BHRS group had lower F/B ratio compared to the T2DM group. Clinical study showed that F to B ratio was increased in T2DM patients compared with the healthy control (Kant et al., 2022). Decreasing the F/B ratio in gut microbiota is beneficial for the treatment of T2DM (Xie et al., 2021). At genus level, the relative abundance of *Candidatus Saccharimonas* and *Ruminococcus* was significantly higher, and the relative abundance of *Lactobacillus*, *Blautia*, *Dubosiella*, and *Anaerostipes* was significantly lower in the T2DM model compared to the control group; after receiving HD-BHRS treatment, the relative abundance of *Lactobaculum*, *Blautia*, and *Anaerostipes* significantly increased, and the relative abundance of *Allobaculum*, *Candidatus Saccharimonas*, and *Ruminococcus* decreased significantly. Many species under the genus *Lactobacillus* have significant effects on glucose metabolism, and several studies have shown that treatment with *Lactobacillus* significantly controls elevated blood glucose and is able to regulate glucose tolerance, hepatic glycogen, and lipid metabolism (Yun et al., 2009; Dang et al., 2018; Zhou et al., 2021). *Blautia* is widely believed to be associated with obesity and inflammation, and it has been demonstrated that *Blautia* reduces obesity and diabetes caused by high-fat diet and relies on its own metabolites, such as acetic acid and SCFA, to alter the intestinal environment and block colonization by specific bacteria (Hosomi et al., 2022). Both *Allobaculum* and *Candidatus Saccharimonas* are known producers of short chain fatty acids (SCFAs), and their elevated abundance affects intestinal pH. It has been shown that these bacteria are positively associated with diabetes and diabetic nephropathy (Lu et al., 2021; Piccolo et al., 2021; Zhao et al., 2021b; Duan et al., 2022). Some SCFA such as butyrate has been demonstrated to enhance the gut permeability (Snelson et al., 2021). Further studies can be carried out to test the effects of BHRS on SCFA levels. The metabolism of sugars and lipids by *Dubosiella* is not well supported by experiments, but we speculate that it may be related to the maintenance of gut microbiota homeostasis. *Ruminococcus* is a large genus that contains both beneficial and harmful bacteria, and [*Ruminococcus*]_*gauvreauii*_group is one of the more important harmful species of *Ruminococcus*, which is positively associated with obesity, hyperlipidemia, and inflammation (Abdel-Daim et al., 2019). *Anaerostipes* is beneficially associated with fasting glucose and estimated glomerular filtration rate (Mazidi et al., 2020; Bui et al., 2021). Likewise, combination treatment of Dan-Lou tablet and Salvia miltiorrhiza ligustrazine could improve dyslipidemia and inhibit inflammatory response in patients through decreasing the abundance of *Anaerostipes* in gut (Zhao et al., 2021). Taken together, our results show that HD-BHRS has a significant ability to correct gut microbiota dysbiosis in T2DM rats.

## Conclusion

**Figure 6:**
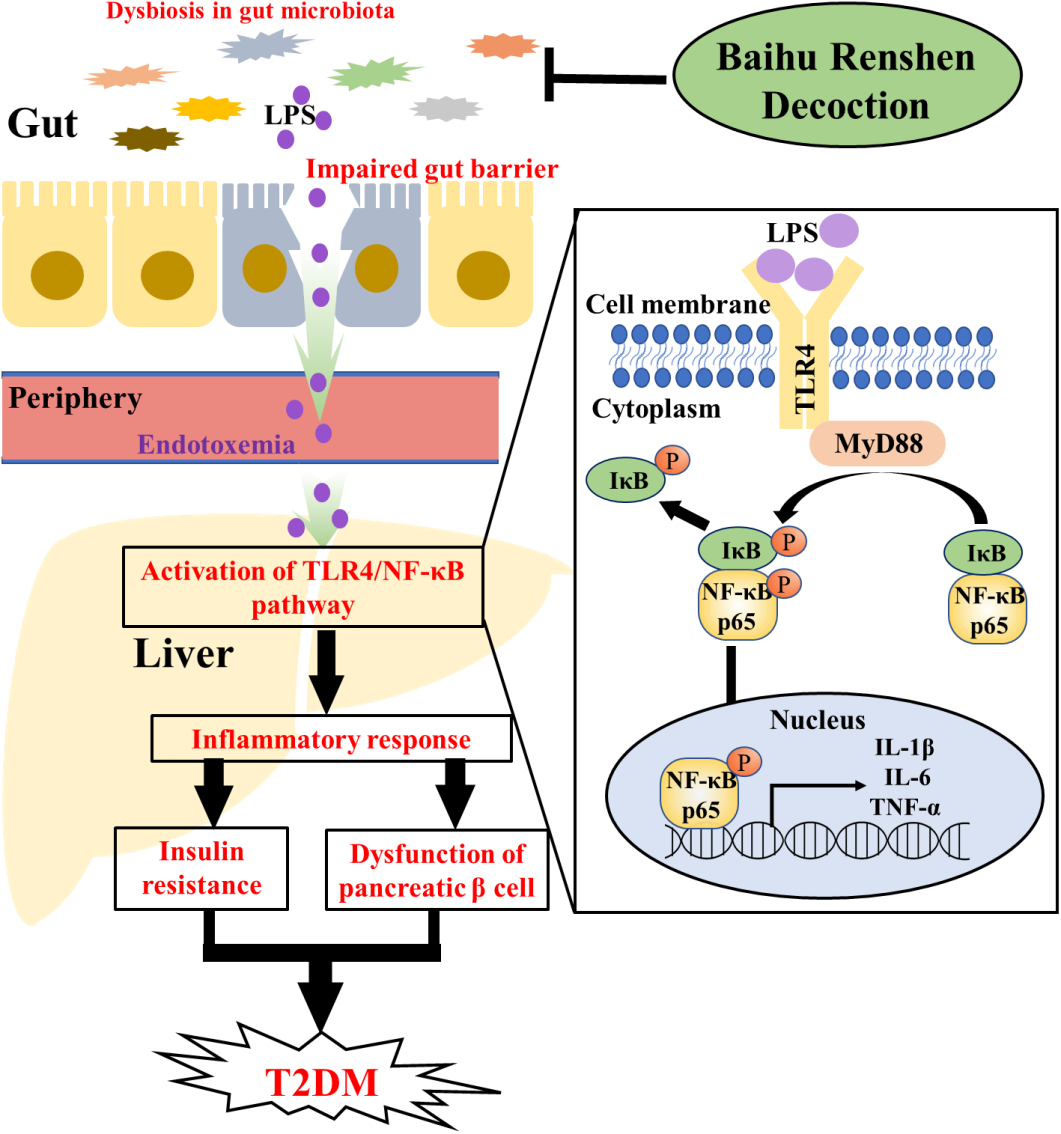
Graphcal abstract. The dysfunction in gut microbiota could impair the tight junction of gut epithelial cells. Then, the LPS of gut microbiota could enter periphery through lack gut barrier and cause endotoxemia. After entering the periphery, LPS could induce inflammatory response in liver through the TLR4/NF-κB pathway [ (1) TLR4 in cellular membrane is activated after bind with LPS; (2) Activated TLR4 recruited MyD88 in cytoplasma to form a TLR4-MyD88 complex; (3) TLR4-MyD88 complex induced the phosphorylation of IκB and NF-κBp65; (4) Phosphorylated NF-κBp65 entered the nucleus and induced the transcription of pro-inflammatory factors such as IL-1β, IL-6 and TNF-α, whereas phosphorylated IκB was degraded in cytoplasma]. The inflammatory response in liver and the released cytokines could contribute to the progression of T2DM through inducing the insulin resistance and the dysfunction of pancreatic β cell. BHRS treatment ameliorated T2DM through regulating the dysbiosis of gut microbiota, enhancing the gut permeability and inhibiting the activation of TLR4/NF-κB pathway.

In conclusion, our study revealed the various ameliorative effects of BHRS on T2DM, including improving the liver and kidney functions and alleviating the hyperglycemia, hyperlipidemia, pathological changes, oxidative stress and inflammatory response. The mechanisms of BHRS on T2DM are likely linked to the repair of gut barrier and the inhibition of TLR4/NF-κB-mediated inflammatory response and the improvement in the dysbiosis of gut microbiota (**Figure 6**).

## Data availability statement

The datasets presented in this study can be found in online repositories. The names of the repository/repositories and accession number(s) can be found in the article/**Supplementary Material**.

## Ethics statement

The animal study was approved by Ethics Committee of Hebei University of Chinese Medicine (Approval no. CZX2021-KY-026).

## Author contributions

BY and BP carried out the experiments and manuscript writing. BY, TT, SZ and WL provided experimental help. XS, Hl and ZZ performed data analysis and result interpretation. YW supervised the experiments. BY and SL provided ideas and technical guidance for the whole work. All authors contributed to the article and approved the submitted version.

## Funding

This work was supported by the Scientific Program Project of Administration of Traditional Chinese Medicine in Hebei Province (No. 2021311), Foundation of Technology Department in Cangzhou (No. 213106042), and Inheritance Studio of Famous Traditional Chinese Medicine Expert Xiuhai Su (2022-75).

## Conflict of interest

The authors declare that the research was conducted in the absence of any commercial or financial relationships that could be construed as a potential conflict of interest.

## Publisher’s note

All claims expressed in this article are solely those of the authors and do not necessarily represent those of their affiliated organizations, or those of the publisher, the editors and the reviewers. Any product that may be evaluated in this article, or claim that may be made by its manufacturer, is not guaranteed or endorsed by the publisher.
