# Supplementary material for: Baihu renshen decoction ameliorates type 2 diabetes mellitus in rats through affecting gut microbiota enhancing gut permeability and inhibiting TLR4/NF-κB-mediated inflammatory response
Source: Front Cell Infect Microbiol. 2022 Nov 11;12:1051962. doi: 10.3389/fcimb.2022.1051962 (PMC9691847; doi:10.3389/fcimb.2022.1051962)
Supplement: Supplementary file 1 [file DataSheet_1.docx]

**Reagents**

High-fat-diet was purchased from Beijing Sibeifu Bioscience Co., Ltd. (Beijing, China). Streptozotocin (STZ) and metformin were purchased from Solarbio Biotechnology Co., Ltd. (Beijing, China). Triglyceride (TG, cat: A110-1-1), total cholesterol (TC, cat: A111-1-1), high density lipoprotein (HDL, cat: A112-1-1), low density lipoprotein (LDL, cat: A113-1-1), alanine aminotransferase (ALT, cat: C009-1-1), aspartate aminotransferase (AST, cat: C0101-2-1), blood urea nitrogen (BUN, cat: C013-2-1), creatinine (Cr, cat: C011-2-1), superoxide dismutase (SOD, cat: A001-3-2), methane dicarboxylic aldehyde (MDA, cat: A003-1-2), and glutathione peroxidase (GSH-Px, cat: A006-2-1) biochemical test kits were obtained from Nanjing Jiancheng Biological Engineering Institute (Nanjing, China). Enzyme-linked immunosorbent assay (ELISA) kits of rat insulin, tumor necrosis factor alpha (TNF-α, cat: m1002859), interleukin (IL)-1β (cat: ml028514), IL-6 (cat: ml102828), Lipopolysaccharide (LPS, cat: ml396700-2) were purchased from Shanghai Enzyme-linked Biotechnology Co., Ltd. (Shanghai, China). ZO-1 (cat:61-7300) and occluding (cat: 71-1500) were purchased from Thermo Fisher Scientific. TLR4 (cat: 19811-1-AP) and MyD88(cat: 67969-1-Ig) were purchased from Proteintech Group, Inc. IκB(cat: ab32518), p-IκB(cat: ab133462) were purchased from Abcam. NF-κBp65(cat: #8242), and p-NF-κBp65(cat: #3033) were purchased from Cell Signaling Technology.

**Quality control of BHRS using ultra performance liquid chromatography (UPLC) coupled with quadrupole-time of-flight (Q-TOF) mass spectrometer (MS) systems**

In order to enhance the reproduction of our study, the main components in BHRS have been detected using ultra performance liquid chromatography (UPLC) coupled with quadrupole-time of-flight (Q-TOF) mass spectrometer (MS) systems based on our previous studies. Briefly, the test solution was injected onto an ACQUITY UPLC BEH C_18_ column (2.1mm×100mm, 1.7μm). The column temperature was 50°C. The injection volumn was 2 μL and the flow rate was 0.3 mL/min and. Mobile phase A was 0.1% formic acid aqueous solution and mobile phase B was acetonitrile contained 0.1% formic acid. The mobile phase conditions were: 0 min, 5% B; 1 min, 10% B; 6 min 60% B; 6.5 min 100% B; 10 min 100% B; 10.1 min 5% B; 13 min 5% B.

A Q-TOF MS equipped with an electrospray ionization (ESI) source was used for negative ionization scan modes (m/z ranges from 50 to 1,200 Da). The scan time was 0.2 s. The capillary voltage was 2,200 V. The desolvation temperature was 350°C and the source temperature was 100℃. The sample cone voltage was 40 V and the extraction cone voltage was 4V. The cone gas flow was 40 L/h and the desolvation gas flow was 800 L/h.

Ginsenoside Rb1 and ginsenoside Re in *Panax ginseng* C.A.Mey., liquiritin in *Glycyrrhiza glabra* L., timosaponin BII in *Anemarrhena asphodeloides* Bunge are used as the reference standards for the quality control of BHRS (Supplementary material). Figure S1 showed the chromatograms of BHRS and the reference standards. The detailed information of these compounds was shown in Table S1.

**a**

**
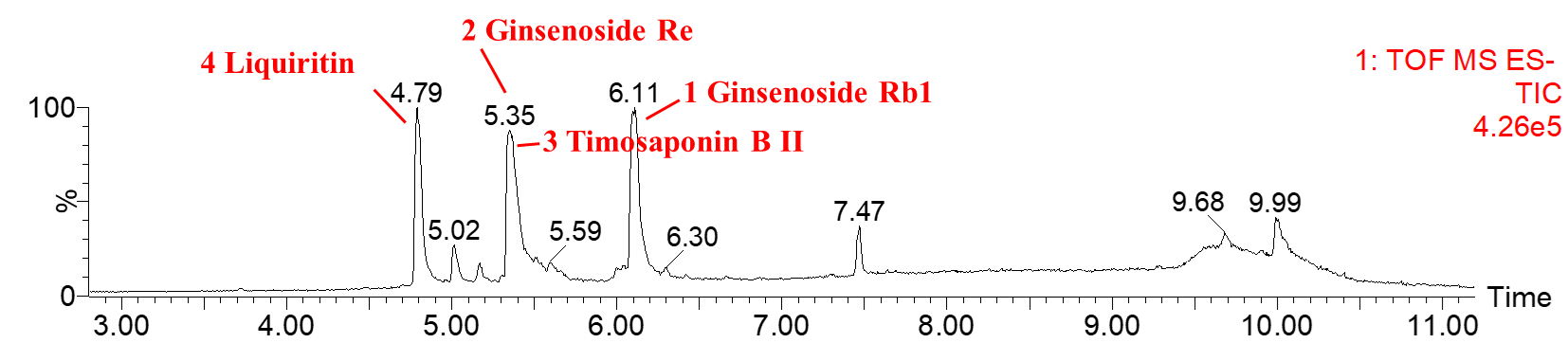
**

**b**

**
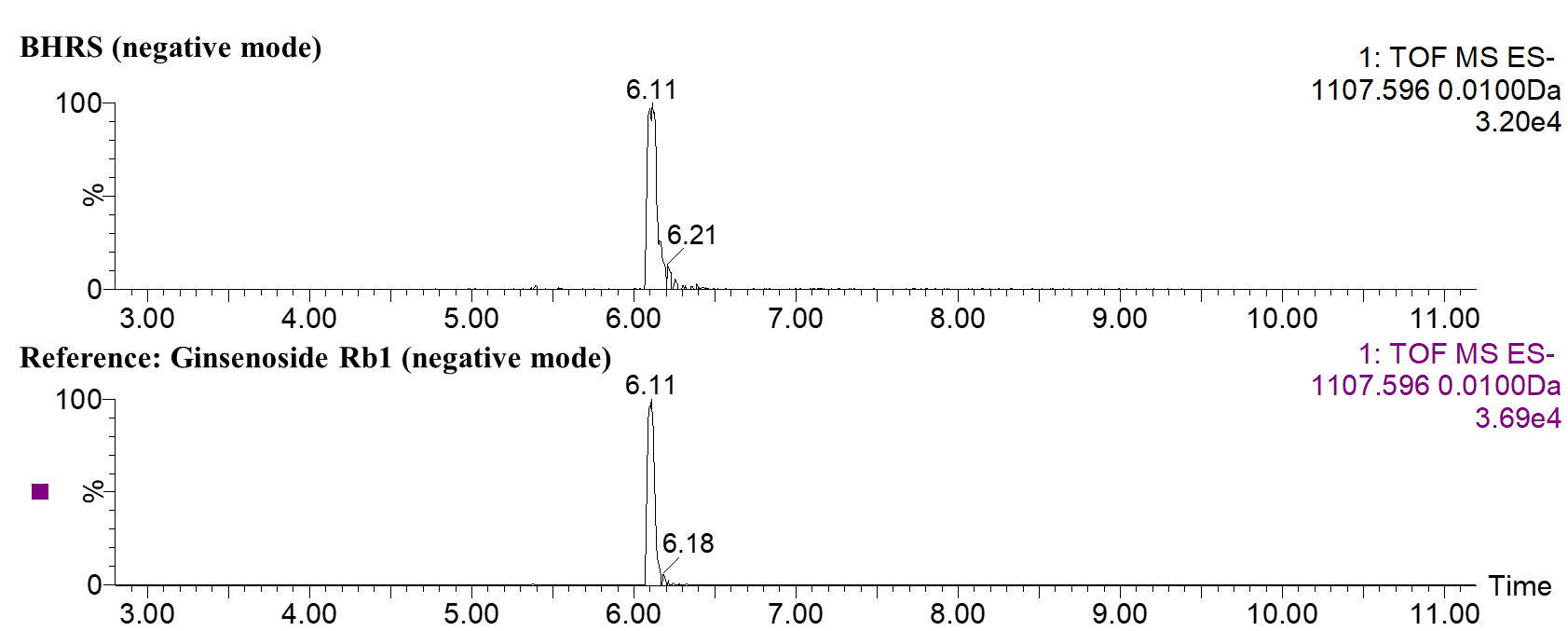
**

**c**

**
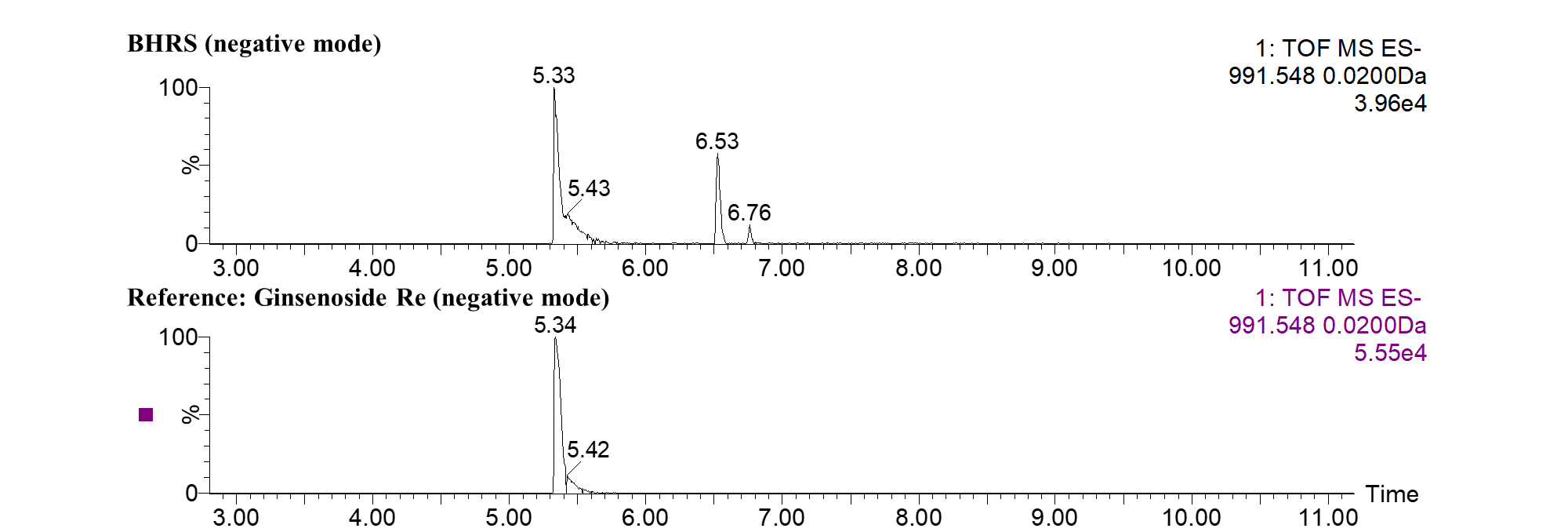
**

**d**

**
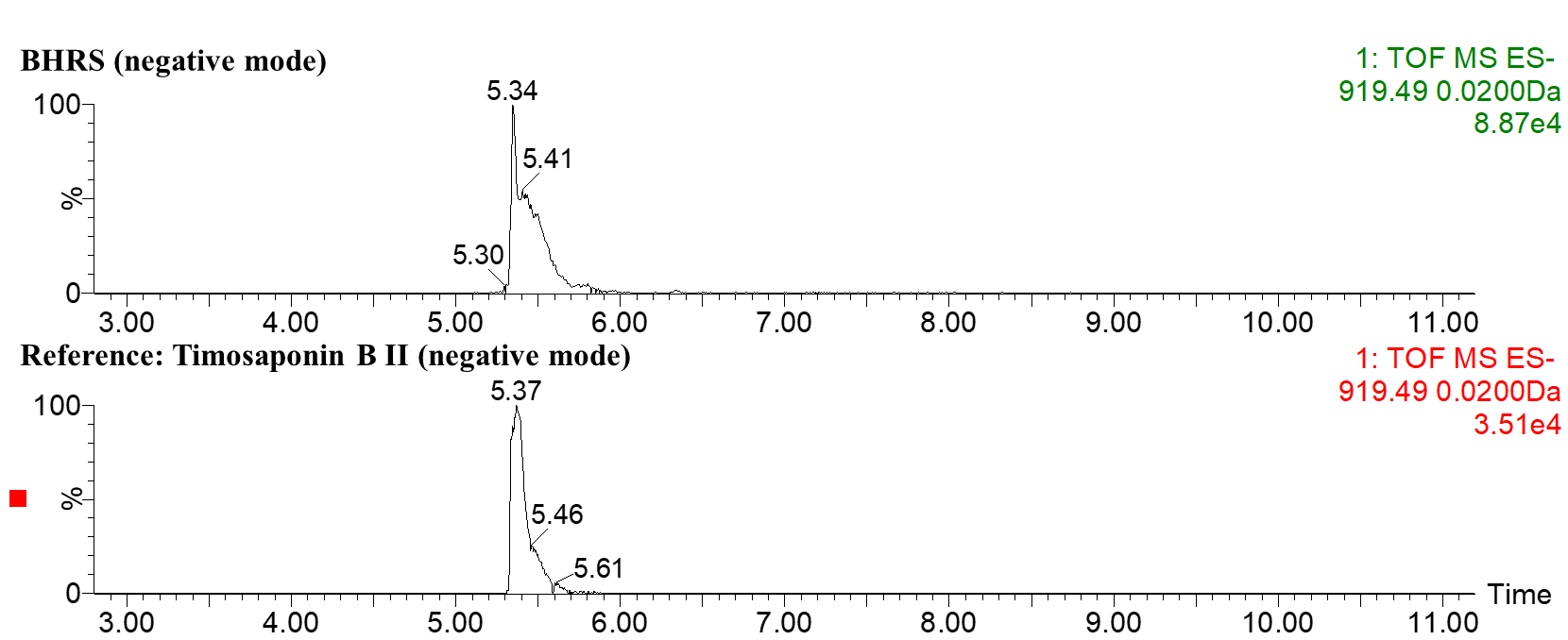
**

**e**

**
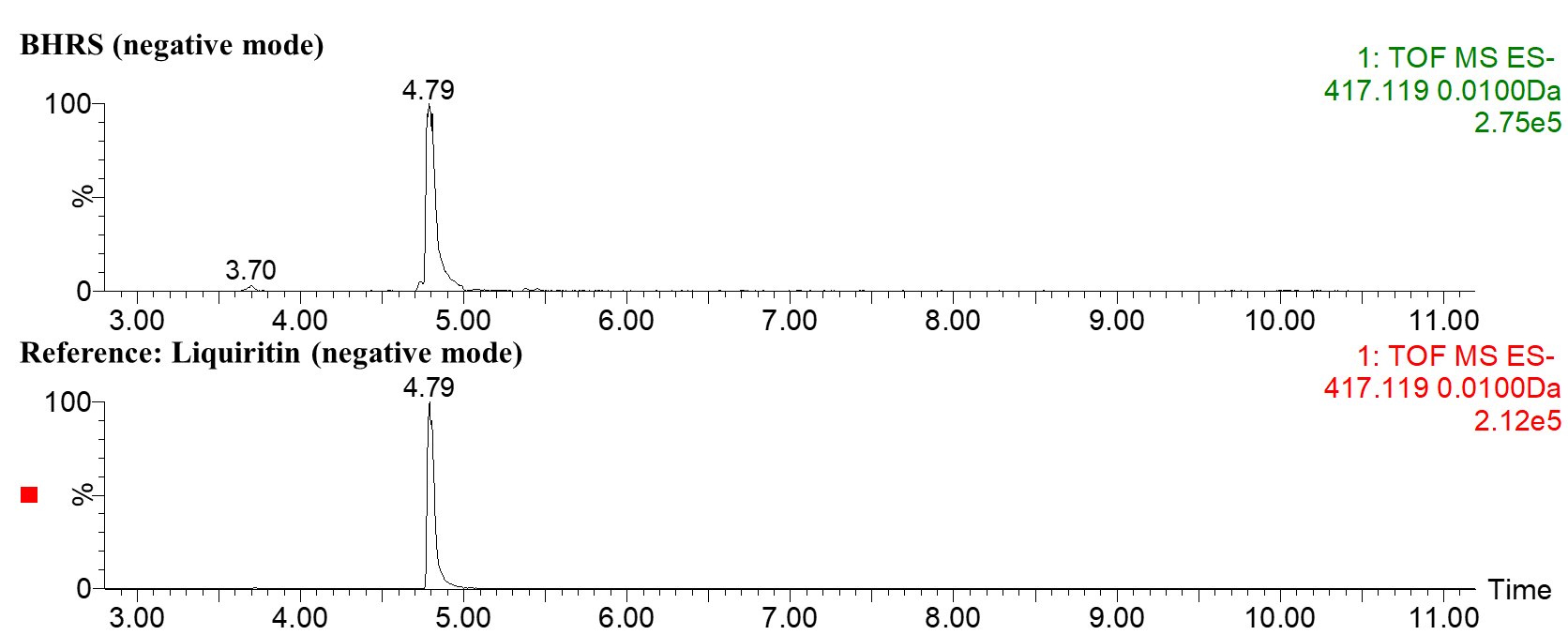
**

**Figure S1:** The chemical profiles of BHRS using UPLC-MS. (**a**) The total ion chromatogram in negative ion modes (**b-e**) The main bioactive compounds of ginsenoside Rb1 (b), ginsenoside Re (c), timosaponin B II (d), liquiritin (e).

**TABLE S1 The characteristic fragment ions of reference standards in BHRS**

| **Marking**  **peak no.** | **Name** | **RT**  **(min)** | **Ion** |
| --- | --- | --- | --- |
| 1 | Ginsenoside Rb1 | 6.11 | [M-H]^-^ |
| 2 | Ginsenoside Re | 5.34 | [M-H]^-^ |
| 3 | Timosaponin B II | 5.37 | [M-H]^-^ |
| 4 | Liquiritin | 4.79 | [M-H]^-^ |

**TABLE S2 Primer sequences**

| **Genes ID** | **Full Name** | **Primer sequence (5’-3’)** |
| --- | --- | --- |
| *β-actin* | Actin, beta | Forward: AGATGACCCAGATCATGTTTGAGA |
|  |  | Reverse: GCATGAGGGAGCGCGTAA |
| *IL-1β* | Interleukin 1 beta | Forward: GGCTGACAGACCCCAAAAGA |
|  |  | Reverse: TGTCGAGATGCTGCTGTGAG |
| *IL-6* | Interleukin 6 | Forward: CACTTCACAAGTCGGAGGCT |
|  |  | Reverse: TCTGACAGTGCATCATCGCT |
| *TNF-α* | Tumor necrosis factor alpha | Forward: ATGGGCTCCCTCTCATCAGT |
|  |  | Reverse: GCTTGGTGGTTTGCTACGAC |
